# Four-dimensional conditional averaging tomography of rotating plasma ejection from cylindrical detached plasma

**DOI:** 10.1038/s41598-024-59182-5

**Published:** 2024-04-23

**Authors:** Hirohiko Tanaka, Shin Kajita, Hiroki Natsume, Noriyasu Ohno

**Affiliations:** 1 Institute of Materials and Systems for Sustainability, Nagoya University, Furo-cho, Chikusa-ku, Nagoya, 464-8603 Japan; 2 Graduate School of Engineering, Nagoya University, Furo-cho, Chikusa-ku, Nagoya, 464-8603 Japan; 3 Graduate School of Frontier Sciences, The University of Tokyo, Kashiwa, Chiba 277-8561 Japan; 4 Global Research Institute of Nuclear Energy, Tokai University, Hiratsuka, 259-1207 Japan

**Keywords:** Plasma physics, Magnetically confined plasmas, Imaging and sensing

## Abstract

Detached plasma formation is a way to reduce the heat load on the wall in magnetic fusion devices. This study proposes a novel analysis technique consisting of the conditional averaging, sliding window, and tomography to reveal the spatiotemporal behavior of the rotating radial ejection event of detached plasma, which further contributes to local heat load reduction. The used equipment is a high-speed camera and an electrostatic probe located at the periphery of the linear plasma device NAGDIS-II. By applying this method, four-dimensional (4D) behavior of the emission structure along time (1D) and space perpendicular and parallel to the magnetic field (3D) was clarified; a rotating distorted structure appears as a precursor, which is then scraped and transported radially and axially. The proposed method is widely applicable to short-term rigid-body rotating structures, especially in linear plasmas.

## Introduction

The detached plasma formation, in which the interactions between plasma and neutral particles promotes volume recombination processes, is inevitable for sufficient reduction of the divertor heat load in future magnetic fusion devices^1^. In addition, an enhancement of the convective plasma transport across the magnetic field is observed in detached plasmas of several magnetic confinement devices, including tokamak^2–5^, helical^6^, and linear devices^7–13^. Such the cross-field transport contributes to the further reduction of the local heat flux, and understanding this physics could lead to a significant reduction of the divertor heat load by enhancing transport.

In the linear plasma devices NAGDIS-II^11^ and Magnum-PSI^13^, previous studies revealed that the enhanced transport occurs near the volume-recombining region called the “recombination front”, and the transport dynamics on the perpendicular cross section of the magnetic field have been fairly understood^9,12,14^. However, the global behavior, including the parallel direction, is not sufficiently clear, and the enhancement mechanism is still unknown.

In order to further elucidate the enhanced cross-field transport in the detached plasmas, it is necessary to investigate the dependence of the global spatiotemporal transport characteristics on various experimental conditions. One measurement method is to use a large number of electrostatic probes, as in the linear device LMD-U^15^. However, it would be difficult to apply this method in detached plasmas, because the heat load is too high near the high-density recombination front, and the state can be strongly perturbed by inserting a large number of probes since the detached plasma is unstable. Using only two electrostatic probes, where the position of one probe is fixed and the other is varied, the spatiotemporal parameter evolution just before and after the plasma ejection event was extracted in NAGDIS-II with minimal disturbance^12^ by applying a statistical technique called the conditional averaging^16,17^. However, this method takes a long time to acquire a large number of data sets so that it is not suitable for parameter scans, and the heat load problem remains.

The use of high-speed cameras is a powerful method for capturing the spatiotemporal evolution. However, one of the limitations is in the fact that the signals obtained are line-integrated and do not reflect local values. To obtain local values from line-integrated signals, tomography is an effective method. In general, there are two types of tomography: those that use detectors with multiple angles of view, and those in which the subject or detector rotates, as in medical tomography called the spiral (helical) computed tomography (CT)^18^. The former has been applied to fluctuations in the linear device PANTA (modified from LMD-U), for example, by arranging multi-channel diode arrays, instead of high-speed cameras, with multiple azimuthal angles of view at several axial locations^19^, but the hurdle for installing such the equipment to achieve both high spatial and temporal resolutions is quite high. In the latter spiral CT case, stationarity is generally required and therefore it cannot obtain time evolution.

Here, we show a novel method to reveal the four-dimensional (4D) distribution of the rotating ejection phenomenon in a cylindrical detached plasma in NAGDIS-II from high-speed camera data viewed from one direction. The method is consisting of the conditional averaging, sliding window, and tomography, and the emission behavior in time (1D) and space perpendicular to and parallel to the magnetic field (3D) was extracted. In this analysis, by assuming rigid-body rotation of the emission structure over short periods during the ejection phase, the camera data can be treated similar to the case where the detector rotates around the object. The equipment used is a high-speed camera and also an electrostatic probe located in the periphery, which eliminates the experimental limitation of heat load.

## Results and discussion

### High-speed camera measurement

**Figure 1:**
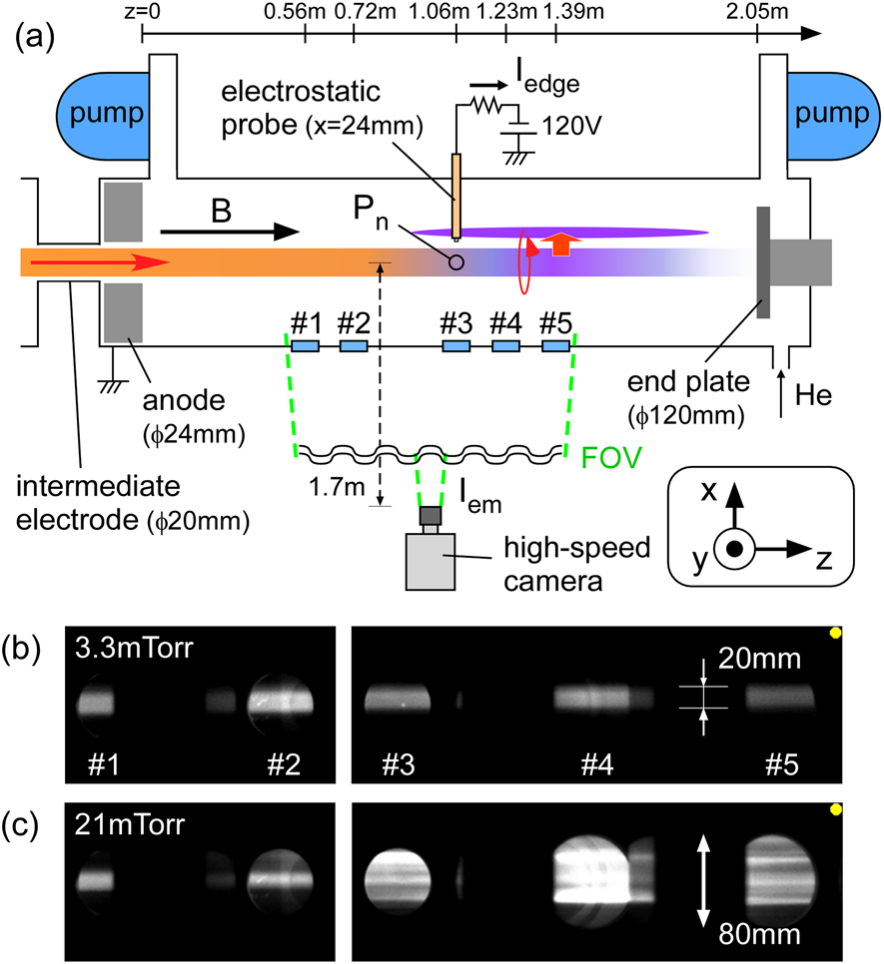
(**a)** Schematic of the experimental setup with the high-speed camera. Pure helium gas was used for the discharge, where the magnetic field strength (*B*), discharge current, and discharge voltage were 0.2 T, 90 A, and 108 V, respectively. Snapshots at (**b**) *P*_n_ = 3.3 mTorr and (**c**) 21 mTorr.

We have used the linear plasma device NAGDIS-II, a high-speed camera, and an electrostatic probe (see “Methods”). By installing the high-speed camera far from the side of the divertor test region (see Fig. 1a), five observation windows (#1, #2, #3, #4, and #5) axially aligned in a length exceeding 0.8 m were set in a field of view (FOV) with high aspect ratio of 20:3. In the FOV, emission along the height (*y*) can be also detected within the window diameter of ~ 55 mm for windows #1–3 and ~ 80 mm for windows #4–5. As a result, visible light emissions from large areas along and across the magnetic field were simultaneously acquired.

Figure 1b is a typical snapshot of the light emissions captured by the high-speed camera in low neutral pressure condition at *P*_n_ = 3.3 mTorr. Here, *P*_n_ is the neutral gas pressure, which was monitored using a capacitance manometer at the same axial position as window #3 at *z* = 1.06 m. Here, *z* is the axial distance from the anode. In this state, an ionizing plasma of approximately 20 mm in diameter is almost uniformly visible in all windows. Because this plasma is terminated by the end plate, this condition is called the “attached state”. The electron temperature *T*_e_, the electron density *n*_e_, and the space potential *V*_s_ measured by the single probe at *z* = 1.06 m (window #3) are 5.7 eV, 3.3 × 10^19^ m^−3^, and − 36 V at the radial center (*x* = 0) and 3.5 eV, 1.7 × 10^19^ m^−3^, and -7.4 V at *x* = 15 mm, respectively. There is a potential well across the magnetic field due to the configuration of the negatively biased cathode and the hollow anode^11^, which causes the radial electric field (*E*_r_) and the azimuthal rotation of the plasma column due to the *E*_r_ × *B* drift.

By increasing neutral pressure in the divertor test region, the plasma in front of the end plate turns into the recombing plasma when *T*_e_ becomes less than ~ 1 eV^11,20^, and the particle flux to the end plate is significantly reduced (called the “detached state”). Figure 1c is a snapshot captured in a detached state at *P*_n_ = 21 mTorr. In this snapshot, an ionizing plasma and a recombining plasma are formed upstream and downstream, respectively, in the high-aspect-ratio FOV. At *z* = 1.06 m, *T*_e_, *n*_e_, and *V*_s_ were 5.4 eV, 3.8 × 10^19^ m^−3^, and -22 V at *x* = 0, and 0.8 eV, 3.6 × 10^19^ m^−3^, and -17 V at *x* = 15 mm, respectively. Therefore, the central ionizing plasma is surrounded by recombining plasma in this location. In the downstream windows (#3–5), plasma emissions are observed that extends vertically beyond the window diameter with filamentary structures which are aligned along the magnetic field, while the emission profile in the upstream windows (#1–2) does not change significantly.

The spatiotemporal behavior of the emission structures due to the radial plasma ejection is the interest of this study. In the following, the normalized emission intensity *I*_em_(*y*, *z*_w_, *t*) (see “Methods”) as functions of height *y* and time *t* at each window location (*z* = *z*_w_) is analyzed. In addition, the electrostatic probe at *z* = 1.06 m is simultaneously used to measure the fluctuation during the high-speed camera measurement. The probe head was located at *x* ~ 24 mm, which is far away from the radial center. This measured an edge ion saturation current (*I*_edge_) fluctuation, which tells us when the high-density plasma structure passes through the probe head.

### Mean and fluctuation distributions

**Figure 2:**
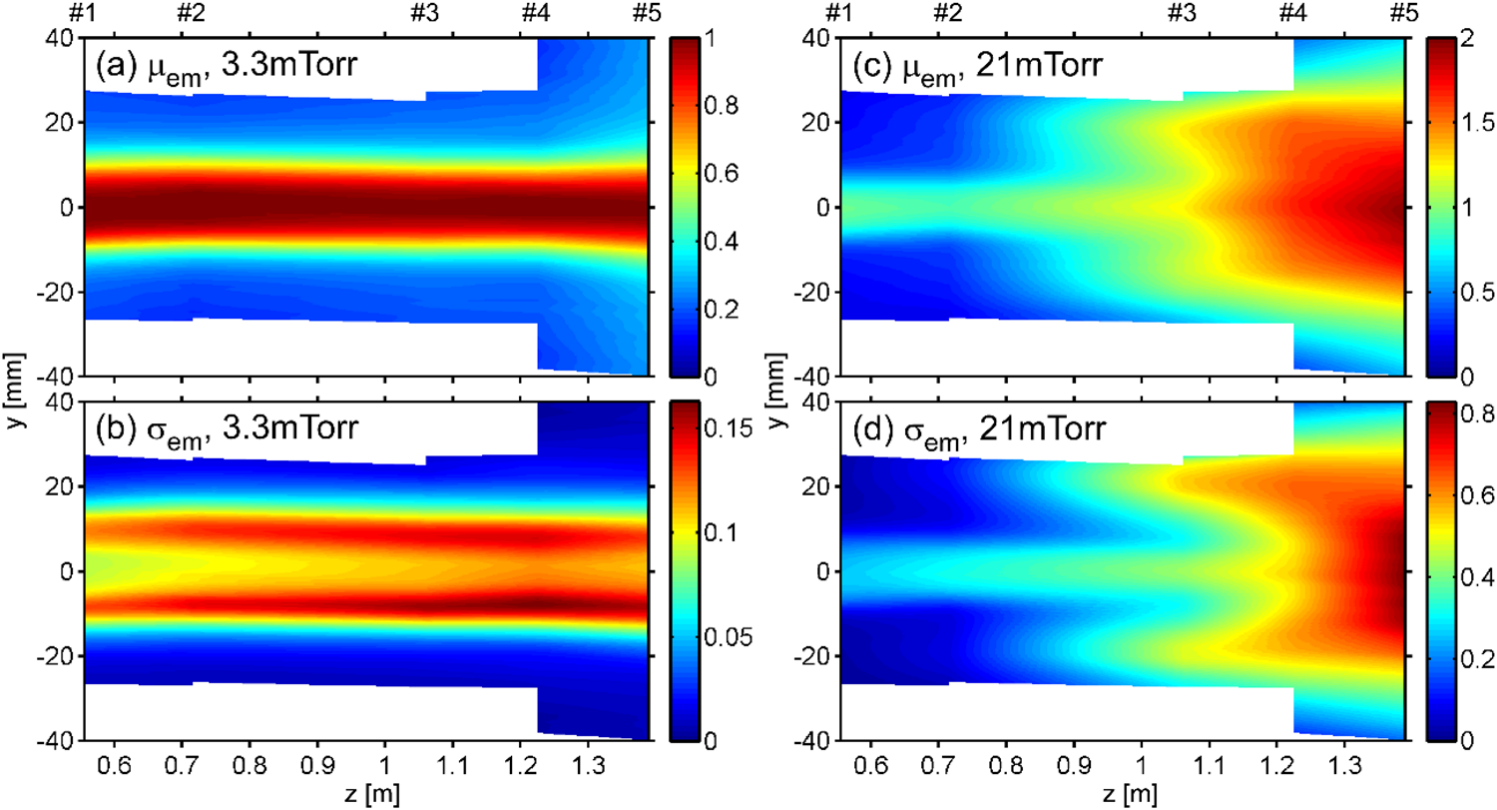
2D images of (**a**) mean (*µ*_em_) and (**b**) standard deviation (*σ*_em_) at *P*_n_ = 3.3 mTorr and (**c**) *µ*_em_ and (**d**) *σ*_em_ at *P*_n_ = 21 mTorr. Note that *µ*_em_ and *σ*_em_ between adjacent windows is linearly interpolated.

Figure 2a shows 2D image of time-averaged *I*_em_, $\mu_{{{\rm{em}}}} \left( {y,z_{{\rm{w}}} } \right) = \langle I_{{{\rm{em}}}}\rangle$, where $\langle\,\rangle$ means time average, as functions of the axial position *z* and height *y* at *P*_n_ = 3.3 mTorr in the attached state. Since there are five measurement windows along the axis, the intensity between adjacent windows is expressed by linear interpolation. The radial profiles of *µ*_em_ at all windows are similar, and its diameter is roughly 20 mm, which is comparable with the intermediate-electrode diameter between the discharge region and the divertor test region. Figure 2b shows the standard deviation of *I*_em_, $\sigma _{\rm{em}} \left( {y,z_{\rm w} } \right) = \langle I_{\rm{em}}^{{2}}\rangle^{0.5}$. Large peaks of *σ*_em_ are observed at *y* =  ± 10 mm where large radial gradients of *µ*_em_ exist. This fluctuation consists of a periodic fluctuation at a frequency of ~ 33 kHz, which would be due to the drift or flute instability that often occurs in linear plasma devices^21^.

Figure 2c shows *µ*_em_ at *P*_n_ = 21 mTorr in the detached state. At the axial position of window #5, *µ*_em_ at *y* = 0 is about twice that at *P*_n_ = 3.3 mTorr. Since *T*_e_ should be lower due to higher *P*_n_, emission from highly excited state atoms generated by the volume recombination processes would be observed. Therefore, the recombination front where the volume recombination dominates^22^ is located at or downstream of window #5. Additionally, it is found that the radial profile significantly broadens around there. At the same location, strong fluctuation appears at *y* ~  ± 20 mm, as shown in the 2D image of *σ*_em_ in Fig. 2d. These features are consistent with previous researches, indicating that the radial ejection locally occurs near the recombination front and radial profile broadens^10,11^. Detailed spatiotemporal behavior of this fluctuation will be shown in the following part.

### Spatiotemporal behavior

**Figure 3:**
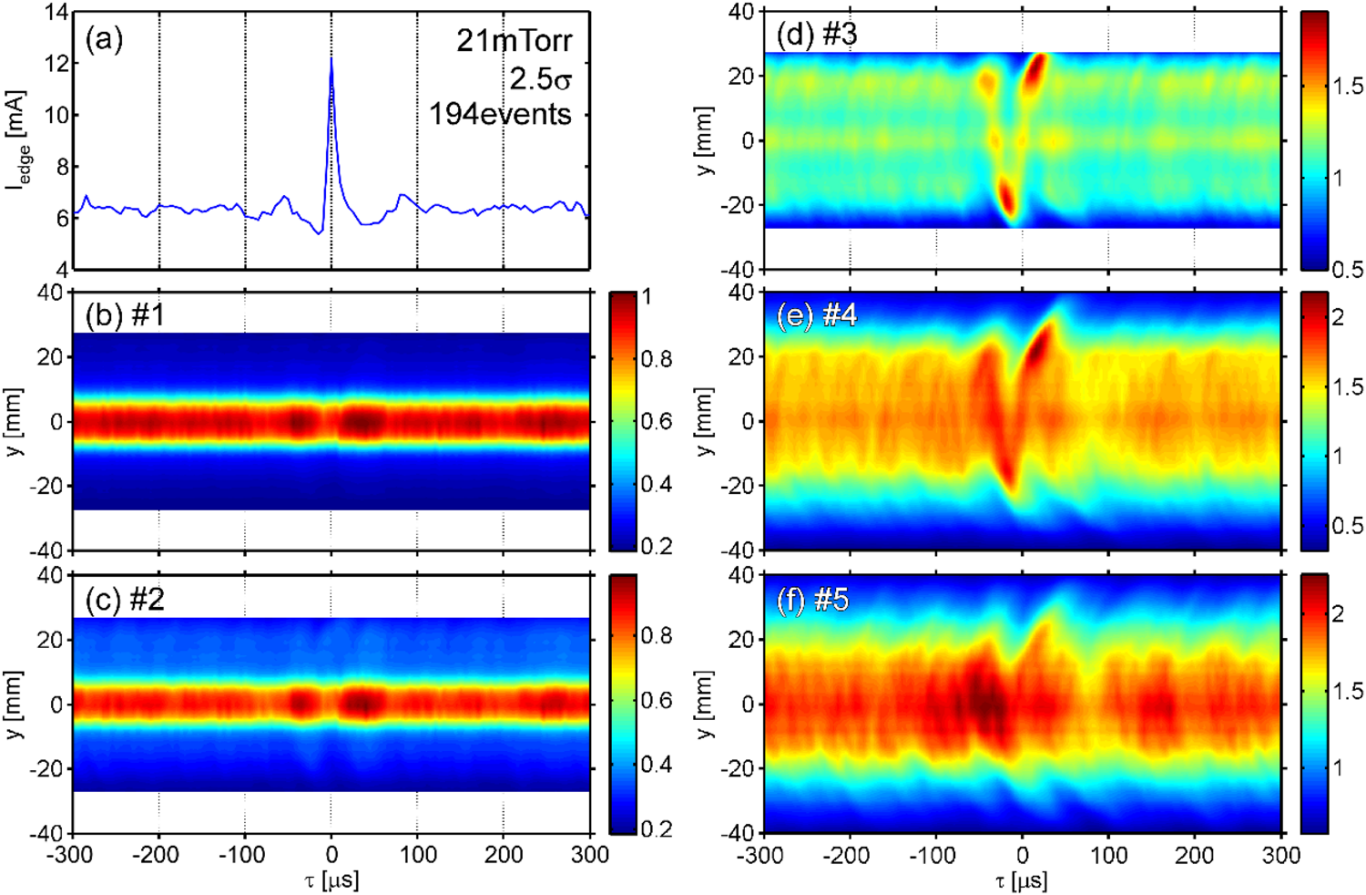
**(a)** Auto-CA shape of *I*_edge_. Cross-CA shapes of *I*_em_ at (**b**) windows #1, (**c**) #2, (**d**) #3, (**e**) #4, and (**f**) #5. *I*_edge_ was used as the reference signal with a threshold of 2.5 times the standard deviation. A total of 194 events were detected and then averaged.

By applying the conditional averaging (CA) analysis (see “Methods”), auto-CA shape of the edge ion saturation current *I*_edge_ at *P*_n_ = 21 mTorr is obtained, as shown in Fig. 3a. It can be seen that *I*_edge_ has one isolated positive spike at *τ* ~ 0. Figure 3b-f shows cross-CA shapes of *I*_em_ at windows #1–5, where *I*_edge_ was used for the reference signal. At windows #1 and #2, emissions are localized around *y* = 0 and slightly modulated in amplitude near the plasma ejection timing. At window #3, on the other hand, the emission broadens in the *y* direction, and V-shaped emission is locally seen around *τ* = 0 in the *τ*-*y* domain. This shape appears due to the line-integral effect when the rotation of *m* = 1 emission structure is viewed from one direction^23^, where *m* is the azimuthal mode number. Thus, the *m* = 1 rotation appears for a short time. From the interval of high-intensity peaks at *y* ~ 20 mm, the rotation period can be known as ~ 70 *µ*s, so that the rotation frequency is *f*_rot_ ~ 14.3 kHz. At window #5, the central emission becomes stronger from *τ* ~ –100 *µ*s and then oscillates in the *y* direction. After the ejection at *τ* ~ 0, the central emission decreases and then returns. The time scale of this central emission change is slower than the rotation. At window #4, behavior with characteristics between windows #3 and #5 is observed.

Now, the rotation period (~ 70 *µ*s) is ~ 14 times the sampling interval of the high-speed camera. Assuming rigid rotation during the half rotation (7 time points), the sliding tomography using a rotating coordinate system was applied (see “Methods” for more detail). The time evolution of the emission behavior obtained at each window is provided in Supplementary Video.

**Figure 4:**
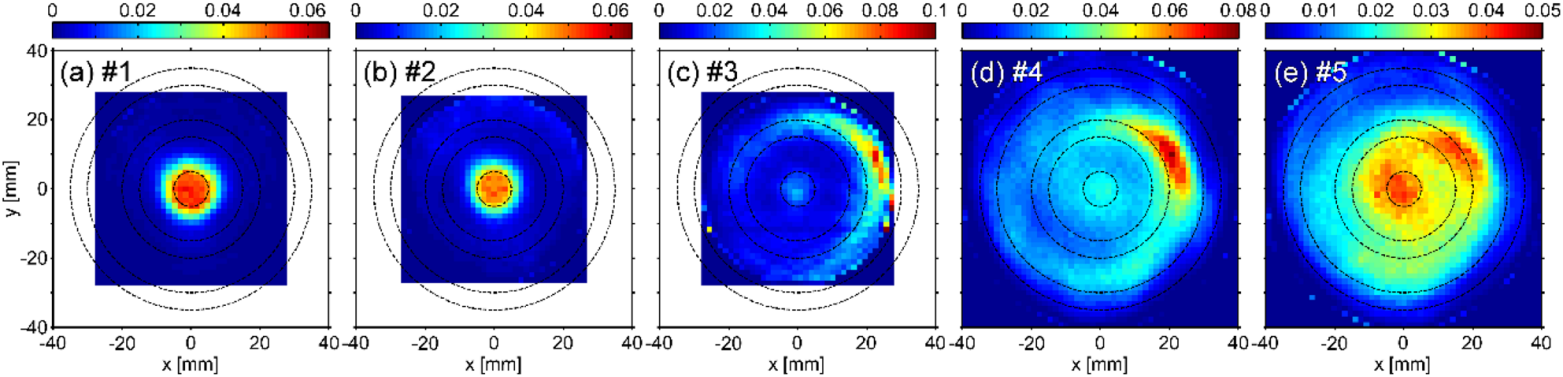
Local emission in the cross-sections perpendicular to the magnetic field at *τ* = 0 for (**a**) windows #1, (**b**) #2, (**c**) #3, (**d**) #4, and (**e**) #5. Dashed lines indicate radial positions with radii of 5, 15, 20, 30, and 35 mm.

Figure 4 shows the local emission distributions (*S*) in the cross sections perpendicular to the magnetic field at *τ* = 0 for windows #1–5. At windows #1–2, similar circular emission distributions can be seen. At the window #3 location, in which the electrostatic probe exists at (*x*, *y*) = (24, 0) mm, it can be seen that strong arc-shaped emission is present near the electrostatic probe. In addition, emission, although weak, is also present in the radial center. Considering the electron temperature described above, edge and central emission can be attributed to the recombining and ionizing plasma components, respectively. At window #4, the arc-shaped structure is more clearly visible at a similar radial and azimuthal position as window #3. Furthermore, at window #5, the central plasma emission is connected to the ejected plasma structure.

**Figure 5:**
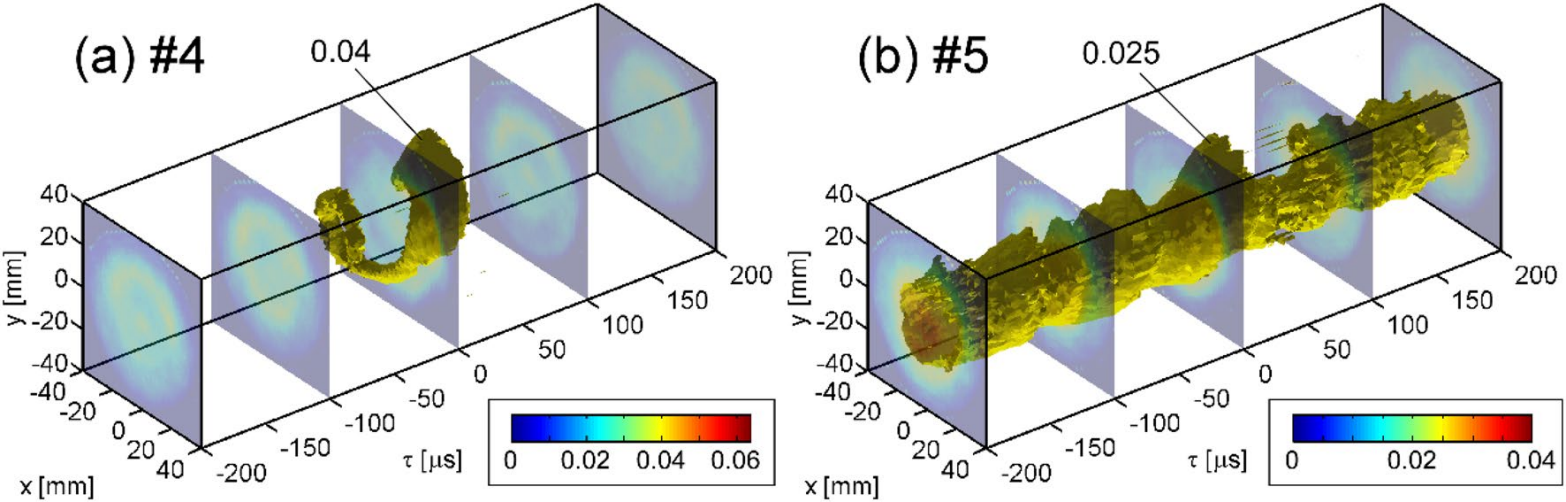
3D displays of time evolutions of local emission on the cross sections at (**a**) windows #4 and (**b**) #5 as functions of *x*, *y*, and *τ*.

Figure 5 shows three-dimensional (3D) displays of time evolutions on the cross-sections at windows #4 and #5. At window #5 (Fig. 5b), after distortion and ejection occurred, the central structure seems to be scraped and then restored. On the other hand, at window #4 (Fig. 5a), the ejected structure appears to be floating. Here, the recombination front, where the radial ejection occurs locally^11^, is located at window #5 or downstream of it, as described above. Furthermore, in the previous study using the Mach probe located at the periphery, plasma flow from the recombination front to the upstream was observed^24^. Therefore, the emission structure floating at window #4 is thought to be the one transported along the magnetic field upstream from the radially ejected plasma structure near the window #5 position.

**Figure 6:**
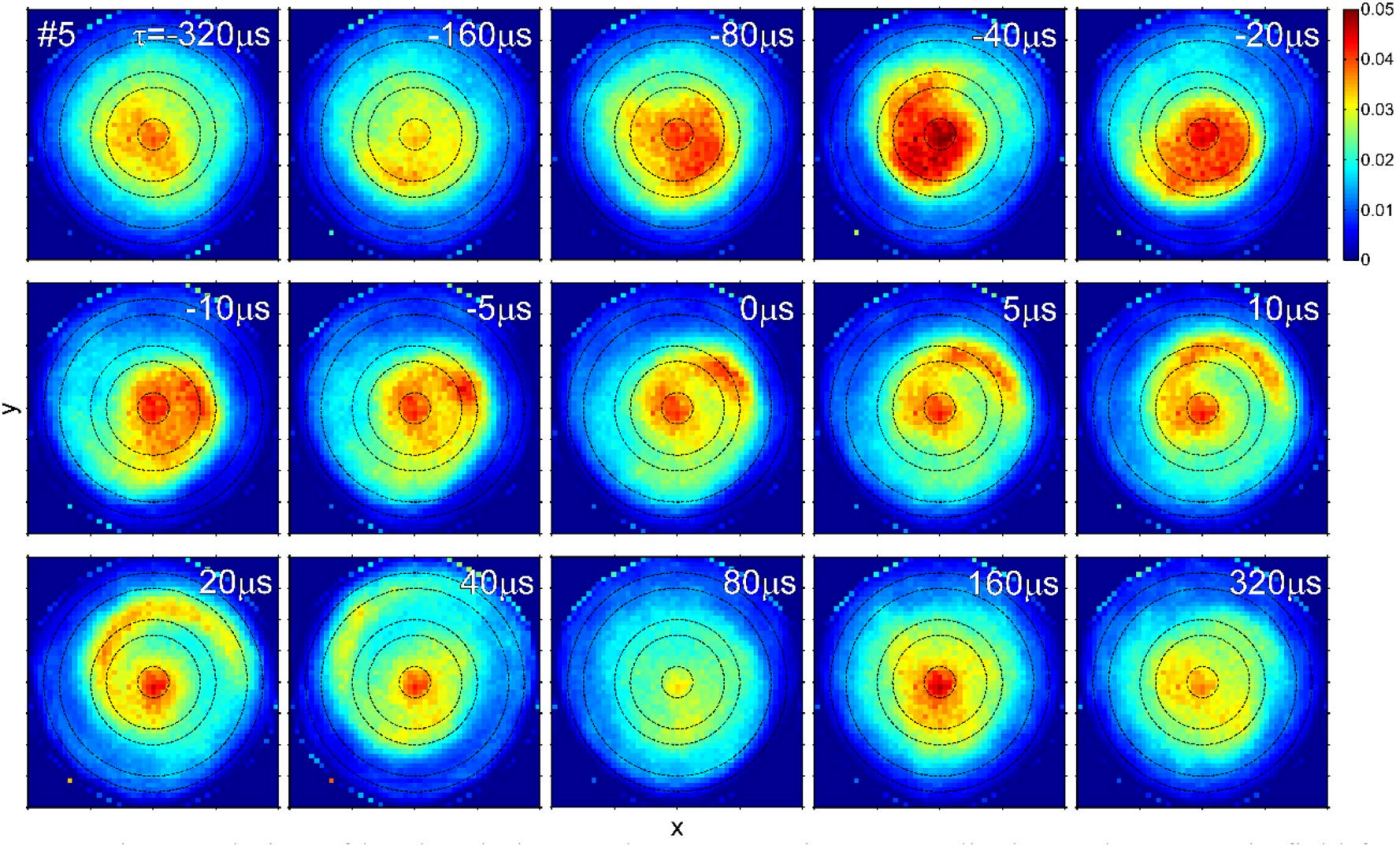
Time evolution of local emission on the cross-section perpendicular to the magnetic field for window #5. Note that the plotted *τ* is selected at finer intervals as it approaches *τ* = 0.

Figure 6 shows the time evolution of the local emission distribution at window #5. It is visualized how the plasma ejection is occurring. Between *τ* = -160 *µ*s and -80 *µ*s, the emission of the plasma center is intensified with *m* = 0. At ~ -80 *µ*s < *τ* <  ~ -10 *µ*s, a distorted *m* = 1 structure that is eccentric from the central axis is rotating. Plasma is then ejected radially from the central region, and the ejection position continues to rotate at ~ -5 *µ*s < *τ* <  ~ 20 *µ*s. As a result, an arc-shaped structure is formed. After that, the ejection stops and the central emission becomes weak at ~ 40 *µ*s < *τ* <  ~ 80 *µ*s, but the emission intensity returns to the original one after a certain period.

Therefore, it is clarified that mode structures with the azimuthal mode numbers of *m* = 0 and 1 appear as precursor events of the radial plasma ejection. Such the behaviors are consistent with the interpretation from the 2D measurement results over radius and time in the previous study^12^. The increase in the central emission just before the radial ejection is attributed to an increase in *n*_e_ and a decrease in *T*_e_, contributing an enhancement of the volume recombination. Thus, there is a possibility that neutral particles produced by the volume recombination enhance the radial transport through the neutral wind effect^16,25^.

## Conclusions

By introducing a simple assumption that the plasma is rotating rigidly for a short time, we have succeeded in obtaining 4D spatiotemporal dynamics (1D in time and 3D in space) from the high-speed camera data viewed from one direction in the linear plasma device NAGDIS-II, which is useful for understanding enhanced cross-field transport in detached plasma. This measurement can be done in a short time, and the setup is simple, using only a high-speed camera and an electrostatic probe for reference signal measurement.

Shortly before the plasma ejection occurs, the central plasma emission intensifies and it makes a rotating distorted structure as precursor, which contains the azimuthal mode numbers of *m* = 0 and 1 components, near the recombination front. In addition, the presence of an isolated emission structure was firstly clarified in the upstream region of the recombination front. An enhanced cross-field transport is expected to broaden the heat flux across the magnetic field and reduce the divertor heat load in fusion devices.

To elucidate the physical mechanism, it would be useful to apply this method to different experimental conditions and study the changes in the spatiotemporal behavior. The obtained spatiotemporal patterns can be further analyzed by applying a mode analysis^8^ etc., and we plan to investigate the axial relationship of the analysis results. Furthermore, since the electrostatic probe for the reference does not need to be inserted into the plasma column, there are no heat load constraints. Thus, this method can be applied to devices with higher heat flux, such as Magnum-PSI^26^. The proposed method is widely applicable to rotating structures and is particularly compatible with linear plasma, which is a self-emitting rotating body, in other fields besides fusion, e.g., in the space propulsion field.

## Methods

### Experimental setup

NAGDIS-II is a linear plasma device consisting of a discharge region and a divertor test region with axial lengths of ~ 0.5 m and ~ 2 m, respectively^27,28^. This device can produce high-density and low-temperature plasmas in steady state by DC arc discharge with a heated LaB_6_ cathode. There is an intermediate electrode with a diameter of 20 mm and an anode with a diameter of 24 mm between the discharge region and the divertor test region. Therefore, in the peripheral region outside the ~ 10 mm radius, the plasma source is not present in the parallel direction and thus plasma is supplied by radial transport.

The pixel size of the applied high-speed camera (nac Image Technology Inc., ACS-1 M60) was 1,280 × 192, leading the width per pixel of approximately 0.77 mm in the current setting. Adjacent windows inside the FOV are spaced approximately 16 cm apart axially, except between two more distant windows #2 and #3. No optical filter was applied. The frame rate was 200 kframe/s and the exposure time was 1 *µ*s. An electrostatic probe made of tungsten was installed in the same axial position as window #3. The sampling frequency of the electrostatic signal was 1 MHz. Simultaneous measurements were made with the camera and electrostatic probe for a time of about 100 ms under each gas pressure condition. The end target, whose diameter is 120 mm and axial position is 2.05 m, was a floating condition electrically.

To handle large movie data *I*_mov_(*y*, *z*, *t*), which is a function of height *y*, axial position *z*, and time *t*, we first extracted the emission signal at the axial position *z* = *z*_w_ of each window and *I*_mov_(*y*, *z*_w_, *t*) was obtained. A finite difference in transmittance exists at different windows. Thus, to eliminate its effect, the emission intensity for each window was normalized using the time-averaged emission at the plasma center (*y* = 0) at *P*_n_ = 3.3 mTorr, as $I_{{{\rm{em}}}} \left( {y,z_{{\rm{w}}} ,t} \right) \equiv I_{{{\rm{mov}}}} \left( {y,z_{{\rm{w}}} ,t} \right)/\langle I_{{{\rm{mov}}}} \left( {0,{ }z_{{\rm{w}}} ,t} \right)|_{{P_{{\rm{n}}} = 3.3{\rm{mTorr}}}}\rangle$. The lowest neutral pressure condition at *P*_n_ = 3.3 mTorr is in the attached state, where the emission intensity is considered to be roughly uniform along the magnetic field. The normalized emission intensity *I*_em_ is used in further analysis in this paper.

As shown in Fig. 1a, the camera viewing angle for each port is different in the *x*–*z* plane. The oblique angle from the *x*-axis is about 14 degrees at window #5. This results in an observation position deviation of ± 10 mm along the magnetic field in the *x* = [-40, 40] mm range. Because the characteristic length of the detached plasma parameter is well over 100 mm^29^, this study assumes that the fluctuation structure is uniform in the range of <  ~ 20 mm along the magnetic field. In addition, the oblique line-of-sight measurement increases the light-path length inside the cylindrical plasma. Thus, the line-integrated emission intensity becomes slightly higher than that measured at the line of sight along the *x*-axis (~ 3% at window #5). This effect is sufficiently small and is canceled out in the normalized emission intensity *I*_em_.

### Conditional averaging

Conditional averaging (CA) is a conventional technique to reveal the temporal characteristics of intermittent phenomena, which is often applied to edge plasma fluctuations^30–32^. This method uses a reference signal *I*_ref_ and other signal *I*_other_. If *I*_ref_ and *I*_other_ are the same, it is called auto CA; if they are different, it is called cross CA. When extracting a typical temporal behavior related to positive-spike event, time points when *I*_ref_ exceeds a threshold set at some multiple of the standard deviation are first detected. Then, subsets of *I*_other_ signal around the detected time points are averaged in a same time domain along *τ*, which is the time lag from the peak amplitude time of the detected event. When *I*_other_ is signals measured at multiple locations, typical behavior over the spatiotemporal domain can be extracted just before and after the intermittent event.

In this study, the edge ion saturation current *I*_edge_ was used as *I*_ref_. For the cross CA with *I*_em_, since the sampling interval of *I*_edge_ is shorter than *I*_em_, the *I*_edge_ signal was linearly interpolated to the sampling time of *I*_em_. *I*_em_ is a function of height *y*, time *t*, and window axial position *z*_w_. Therefore, the cross CA provides a spatiotemporal behavior over time lag (*τ*) and height (*y*) at each window position. The statistical averaging process in the CA method reduces noise, which contributes to the subsequent tomographic analysis.

### Sliding tomography for rigid body rotation

The conditionally averaged emission signal within a moving window of finite time width of 2*n*Δ*t* was used. Here, *n* is a natural constant and Δ*t* is the sampling interval of the emission signal, i.e., the reciprocal of the frame rate. Within the time period of *t*_w_ = [*t*_0_–*n*Δ*t*, *t*_0_ + *n*Δ*t*] including the central time *t*_0_, the emission structure is assumed to rotate rigidly in the azimuthal direction at a constant frequency of *f*_rot_. In this case, there are (2*n* + 1) time signals obtained from a fixed azimuthal angle *θ*_0_ in the time period *t*_w_, and it is equivalent to observing a stationary emission structure from (2*n* + 1) azimuthal angles in a rotational coordinate system with a frequency of *f*_rot_. In the analysis of the line-integrated signal, the rotation direction of this rotational coordinate system is arbitrary; therefore, the rotation direction must be determined from the physical background and/or previous researches. The (2*n* + 1) emission signals are combined and then applied to the tomography with the detection probability matrix, taking into account the azimuthal angles in the rotational coordinate system. As a result, reconstruction of local emission at time *t*_0_ is performed. By sliding the window, the time evolution of the reconstruction result can be obtained every sampling interval.

In the linear plasma device NAGDIS-II, the high-speed camera measurement and the orthogonal decomposition showed that the emission structure across the magnetic field is rigidly rotating during the radial ejection event^9,14^. Furthermore, the rotation frequency of the edge plasma was shown to be axially constant, even though the radial electric field *E*_r_ and its induced *E*_r_ × *B* drift speed were not axially constant^33^. This is due to the fact that the plasma is ejected radially from a localized location in the axial direction^11^, and the frequency of the rigid body rotation is determined by the rotation frequency of the location where the ejection occurs. Therefore, the assumption of the rigid body rotation is valid for the ejected plasma structure in this study.

This study applied the sliding tomography using a rotating coordinate system with the following MLEM algorithm. As a line-integrated signal (*I*) for tomography, a minimum value over the cross-CA emission at each window (*I*_em_|_min_) was subtracted because there is a finite emission intensity due to reflections from the wall. In addition, an extremely small value (floating-point arithmetic: *ε* ~ 2.22 × 10^–16^) was then added to avoid division by zero in the tomography calculation, as *I* = *I*_em_–*I*_em_|_min_ + *ε*. It should be noted that window #3 has a wide emission distribution in the *y* direction but a narrow window width, overestimating the reflected emission. The rotation direction was determined from the *E*_r_ × *B* drift direction.

### MLEM algorithm

From a number of tomography methods, the maximum-likelihood expectation–maximization (MLEM) algorithm^34^ was applied based on the previous researches in the linear plasma device PANTA^19,35^. In Ref^35^, several tomography algorithms were considered, and the MLEM was selected for the analysis of plasma turbulence measured with the multi-channel diode arrays; MLEM does not require any priori assumptions unlike Fourier–Bessel-expansion^36^ etc., and showed better results than the algebraic reconstruction technique (ART)^37^. The fluctuating structures to be reconstructed and data features in this study are roughly similar to those in PANTA; therefore, MLEM was selected as the method that would provide a reasonable solution.

From line-integrated signals, *I* = {*I*_1_, *I*_2_, …, *I*_N_}, acquired by *N* sensors, a local signal on *j*-th cell, *s*_*j*_, can be reconstructed by the following iteration calculation:

$$s_{j}^{k + 1} = \frac{{s_{j}^{k} }}{{\mathop \sum \nolimits_{i = 1}^{N} C_{ij} }}\mathop \sum \limits_{i = 1}^{N} \frac{{I_{i} C_{ij} }}{{\mathop \sum \nolimits_{{j^{\prime} = 1}}^{M} C_{{ij^{\prime}}} s_{{j^{\prime}}}^{k} }},$$

where *k* is the iteration number, *M* is the number of cells for which the local signal is computed, and *C*_*ij*_ is the detection probability from *j*-th cell to *i*-th sensor. In this study, to artificially reduce the reconstructed signal outside the observation area, 1 is substituted to *C*_*ij*_ when *j*-th cell is outside the window radius, i.e., 100% of the emission outside the window radius is assumed to be detected by all sensors. After the iteration calculation, the signal per unit area is calculated by *S*_*j*_ = *s*_*j*_/*A*_*j*_, where *A*_*j*_ is the area of *j*-th cell and *S* = {*S*_1_, *S*_2_, …, *S*_M_}.

## Supplementary Information

Supplementary Video 1.

## Data Availability

Raw data were generated at NAGDIS-II. Derived data supporting the findings of this study are available from the corresponding author upon reasonable request.
